# Anesthesia modality does not affect clinical outcomes of intra-arterial vasodilator treatment in patients with symptomatic cerebral vasospasms

**DOI:** 10.12688/f1000research.52324.1

**Published:** 2021-05-25

**Authors:** Corinne Fischer, Sonja Vulcu, Johannes Goldberg, Franca Wagner, Belén Rodriguez, Nicole Söll, Pasquale Mordasini, Matthias Haenggi, Joerg C. Schefold, Andreas Raabe, Werner J. Z'Graggen

**Affiliations:** 1 Department of Neurosurgery, Inselspital, University Hospital Bern, Bern, 3010, Switzerland; 2 University Institute for Diagnostic and Interventional Neuroradiology, Inselspital, University Hospital Bern, Bern, 3010, Switzerland; 3 Department of Intensive Care Medicine, Inselspital, University Hospital Bern, Bern, 3010, Switzerland

**Keywords:** aneurysmal subarachnoid hemorrhage, Nimodipine, Papaverine, delayed cerebral ischemia, general anesthesia, conscious sedation, functional outcome, hypoperfusion

## Abstract

**Background:** Delayed cerebral ischemia and cerebral vasospasm remain the leading causes of poor outcome in survivors of aneurysmal subarachnoid hemorrhage. Refractory cerebral vasospasms can be treated with endovascular vasodilator therapy, which can either be performed in conscious sedation or general anesthesia. The aim of this study is to compare the effect of the anesthesia modality on long-term clinical outcomes in patients undergoing endovascular vasodilator therapy due to cerebral vasospasm and hypoperfusion.

**Methods:** Modified Rankin Scale (mRS) scores were retrospectively analyzed at time of discharge from the hospital and six months after aneurysmal subarachnoid hemorrhage. Additionally, National Institutes of Health Stroke Scale (NIHSS) was assessed 24 hours before, immediately before, immediately after, and 24 hours after endovascular vasodilator therapy, and at discharge and six months. Interventional parameters such as duration of intervention, choice and dosage of vasodilator and number of arteries treated were also recorded.

**Results: **A total of 98 patients were included in this analysis and separated into patients who had interventions in conscious sedation, general anesthesia and a mix of both. Neither mRS at discharge nor at six months showed a significant difference for functionally independent outcomes (mRS 0-2) between groups. NIHSS before endovascular vasodilator therapy was significantly higher in patients receiving interventions in general anesthesia but did not differ anymore between groups six months after the initial bleed.

**Conclusion:** This study did not observe a difference in outcome whether patients underwent endovascular vasodilator therapy in general anesthesia or conscious sedation for refractory cerebral vasospasms. Hence, the choice should be made for each patient individually.

## Introduction

Cerebral vasospasms (CVS) and delayed cerebral ischemia still remain among the leading causes of morbidity and mortality in survivors of aneurysmal subarachnoid hemorrhage (aSAH). Up to 40% of aSAH patients experience symptomatic CVS, resulting in disability in up to 50% thereof. ^1^ CVS, a narrowing of cerebral arteries thought to be caused by blood breakdown products, mostly develop between 5 to 14 days after aSAH. ^2^ So far, there is no therapy known, which was shown in randomized trials to improve cerebral perfusion and thus to avoid brain ischemia and infarction in symptomatic patients. Commonly used rescue treatments for symptomatic CVS include induced hypertension, and in refractory CVS angioplasty or intra-arterial application of vasodilators, e.g. nimodipine or papaverine. ^1, 3, 4^ Both have been shown in case series to improve neurological outcome in said patients. ^5– 7^

In recent years, several studies investigated the best method of anesthesia for endovascular treatment in acute ischemic stroke. ^8– 20^ While initially in mostly retrospective studies, data showed conscious sedation (CS) to be superior, ^8, 9^ a recent meta-analysis showed no significant difference in outcomes for CS and general anesthesia (GA) ^17^ if only randomized controlled trials were considered. ^15, 16, 18^ To the best of our knowledge, no studies comparing CS and GA in endovascular treatments for refractory CVS after aSAH have been performed.

The aim of this study is to compare six-month outcomes for choice of sedation in patients treated with endovascular vasodilators for CVS after aSAH.

## Methods

This is a single-center retrospective case-control study analyzing clinical outcomes in patients with symptomatic CVS after aSAH treated with endovascular vasodilators at the University Hospital Bern, Bern, Switzerland.

### Study design

The University Hospital Bern conducts a prospective database for patients treated with aSAH. This database was retrospectively searched for patients hospitalized between September 2011 and October 2019. Only patients aged >18 and <85 years were included. Inclusion criteria were: 1) aSAH of all severities (World Federation of Neurosurgeons (WFNS) score I–V), 2) secured aneurysm either by endovascular or surgical treatment, 3) refractory CVS treated by intra-arterial admission of either nimodipine and/or papaverine. Exclusion criteria were: 1) incomplete data, 2) loss of follow up, 3) continuous intra-arterial nimodipine treatment, 4) re-rupture of aneurysm during the hospital stay.

Patients were divided into three treatment groups: patients who underwent treatment with endovascular vasodilators in CS only (“CS”), in GA only (“GA”), or patients who received intra-arterial treatments in CS and GA (“both”).

### Data collection

All data was acquired from patient records and the institutional electronic Patient Data Management System (Centricity ^TM^ Critical Care, General Electric Company, GE Healthcare, United States of America). Vital signs are automatically recorded and the bedside team additionally enters clinical scores and administered drugs into the system.

The primary endpoint of this study was functional outcome at six months, analyzed by the modified Rankin Scale (mRS). Secondary outcome parameters included mRS at discharge and National Institutes of Health Stroke Scale (NIHSS) assessed 24 hours before the (first) intra-arterial vasodilator treatment (t _1_), directly before (t _2_), directly after (last) treatment (t _3_), 24 hours after (last) treatment (t _4_) and consecutively at discharge from the hospital (t _5_) and after six months (t _6_). Further parameters consisted of interventional parameters such as duration of intervention, choice of vasodilator (nimodipine or papaverine), number of treated arteries and vasodilator dosage.

Patient characteristics such as age, sex, aneurysm location and treatment and Barrow Neurological Institute (BNI), Fisher, Hunt & Hess and WFNS scores were obtained from institutional patient records.

### Statistical analysis

The statistical analysis was performed using SPSS Statistics 21.0 (IBM, Armonk, NY, USA). The Shapiro-Wilk normality test was used to test for normal distribution.

Univariate Analysis of Variance (ANOVA) test was used to compare “CS”, “GA” and “both” groups for differences in age. Differences in sex and aneurysm treatment were explored with Pearson Chi Squared analysis.

For mRS at discharge and six months, outcomes were divided in functionally independent (mRS 0-2) and functionally dependent (mRS 3-6). A Chi Squared test was used to test for significant group differences between CS, GA and both. An additional subgroup analysis was performed using a Chi Squared test with “CS” and “GA” groups divided into single versus multiple interventions, resulting in five groups (“single CS”, “single GA”, “multiple CS”, “multiple GA”, “both”).

For the analysis of NIHSS, a 3 × 6 analysis of variance (ANOVA) for repeated measures with post hoc Bonferroni correction for multiple comparisons was conducted. The factors were (i) treatment (“CS”, “GA” and “both”) and (ii) time (t _1_ – t _6_).

Interventional parameters were analyzed for each intervention separately and therefore compared between those performed in CS and GA. For the duration of the intervention, a Welch's two sample t-test was performed. The choice of vasodilator was analyzed by Pearson Chi Squared test. Vasodilator dosage as well as number of treated arteries were analyzed with an independent samples t-test.

Data are presented as mean with standard deviation (SD) in brackets and in figures as mean with +1 SD as error bars. A p-value of p < 0.05 was considered statistically significant.

### Ethics statement

This study was carried out in accordance with the recommendations of the local ethics committee (Kantonale Ethikkommission Bern, Switzerland). All subjects gave written general consent in accordance with the Declaration of Helsinki. The protocol was approved by the local ethics committee (Kantonale Ethikkommission Bern, Switzerland).

## Results

In total, 109 patients with refractory CVS treated by intra-arterial admission of either nimodipine and/or papaverine between September 2011 and October 2019 at the University Hospital Bern, Bern, Switzerland were included. Of those, 11 patients had to be excluded. Reasons for exclusion were incomplete data (n = 2), re-rupture of aneurysm during the hospital stay (n = 2), continuous intra-arterial nimodipine treatment (n = 1) and loss of follow-up at six months (n = 6). The final study population consisted of 98 patients, 23 patients in the “CS” group, 53 patients in the “GA” group and 22 patients in the “both” group. In the “CS” group, 16 patients received a single intervention (“single CS”) and seven patients received up to five interventions (“multiple CS”). In the GA group, 26 patients received one intervention (“single GA”) and 27 patients received 2-10 treatments (“multiple GA”). As per definition, all patients in the “both” group received more than one and up to 10 interventions.

### Patient characteristics

Table 1 shows patient characteristics of the three anesthesia groups. Overall mean age was 54.7 years (range 24-81). All groups showed higher percentages of female patients. Age, sex, aneurysm treatment modality did not significantly differ between the three groups.

**Table 1.** Patient characteristics.

	CS	GA	Both	Total	p-value
**Number of patients**	23	53	22	98	
**Mean age** (years, range)	57.0 (32 – 74)	54.0 (24 – 81)	53.7 (36 – 72)	54.7 (24 – 81)	0.550 ^#^
**Sex**					0.219 ^+^
Female	20 (87%)	40 (75%)	21 (91%)	80 (82%)	
Male	3 (13%)	13 (25%)	2 (9%)	18 (18%)	
**Admission WFNS Score**					
I	12 (52%)	15 (28%)	8 (36%)	35 (36%)	
II	1 (4%)	7 (13%)	9 (41%)	17 (17%)	
III	3 (13%)	5 (9%)	1 (5%)	9 (9%)	
IV	4 (17%)	14 (26%)	4 (18%)	22 (22%)	
V	3 (13%)	12 (23%)	0 (0%)	15 (15%)	
**Hunt & Hess Score**					
1	3 (13%)	6 (11%)	4 (18%)	13 (13%)	
2	11 (48%)	16 (30%)	13 (59%)	40 (40%)	
3	3 (13%)	7 (13%)	2 (9%)	12 (12%)	
4	0 (0%)	7 (13%)	1 (5%)	8 (8%)	
5	6 (26%)	17 (32%)	2 (9%)	25 (26%)	
**BNI Score**					
1	0 (0%)	0 (0%)	0 (0%)	0 (0%)	
2	7 (30%)	15 (28%)	8 (36%)	30 (31%)	
3	8 (35%)	22 (42%)	7 (32%)	37 (38%)	
4	5 (22%)	8 (15%)	2 (9%)	15 (15%)	
5	3 (13%)	8 (15%)	5 (23%)	16 (16%)	
**Fisher score**					
1	0 (0%)	0 (0%)	0 (0%)	0 (0%)	
2	2 (9%)	0 (0%)	0 (0%)	2 (2%)	
3	14 (61%)	38 (72%)	18 (82%)	70 (71%)	
4	7 (30%)	15 (28%)	4 (18%)	26 (27%)	
**Aneurysm location**					
Choroideal artery	0 (0%)	1 (2%)	0 (0%)	1 (1%)	
ACA	1 (4%)	0 0%)	0 (0%)	1 (1%)	
ACOM	9 (39%)	18 (34%)	5 (23%)	32 (33%)	
Basilar	2 (9%)	4 (7%)	2 (9%)	8 (8%)	
ICA	1 (4%)	6 (11%)	1 (5%)	8 (8%)	
MCA	5 (21%)	9 (17%)	6 (27%)	20 (20%)	
PCA	1 (4%)	0 (0%)	0 (0%)	1 (1%)	
PCOM	1 (4%)	10 (19%)	7 (32%)	18 (18%)	
A.pericallosa (A2)	2 (9%)	0 (0%)	0 (0%)	2 (2%)	
PICA	0 (0%)	3 (6%)	0 (0%)	3 (3%)	
Superior cerebellar artery	1 (4%)	1 (2%)	0 (0%)	2 (2%)	
Vertebral	0 (0%)	1 (2%)	1 (5%)	2 (2%)	
**Aneurysm treatment**					0.916 ^+^
Clipping	5 (21%)	10 (19%)	4 (18%)	19 (19%)	
Coiling	18 (78%)	42 (79%)	18 (82%)	78 (80%)	
Flow diverter	0 (0%)	1 (2%)	0 (0%)	1 (1%)	

Where not stated otherwise, values represent the number of patients with their respective percentages in brackets. WFNS: World Federation of Neurological Surgeons Score; CS: conscious sedation; GA: general anesthesia.

^#^ univariate ANOVA.

^+^ Chi-squared test.

### Primary outcome

**Figure 1.**
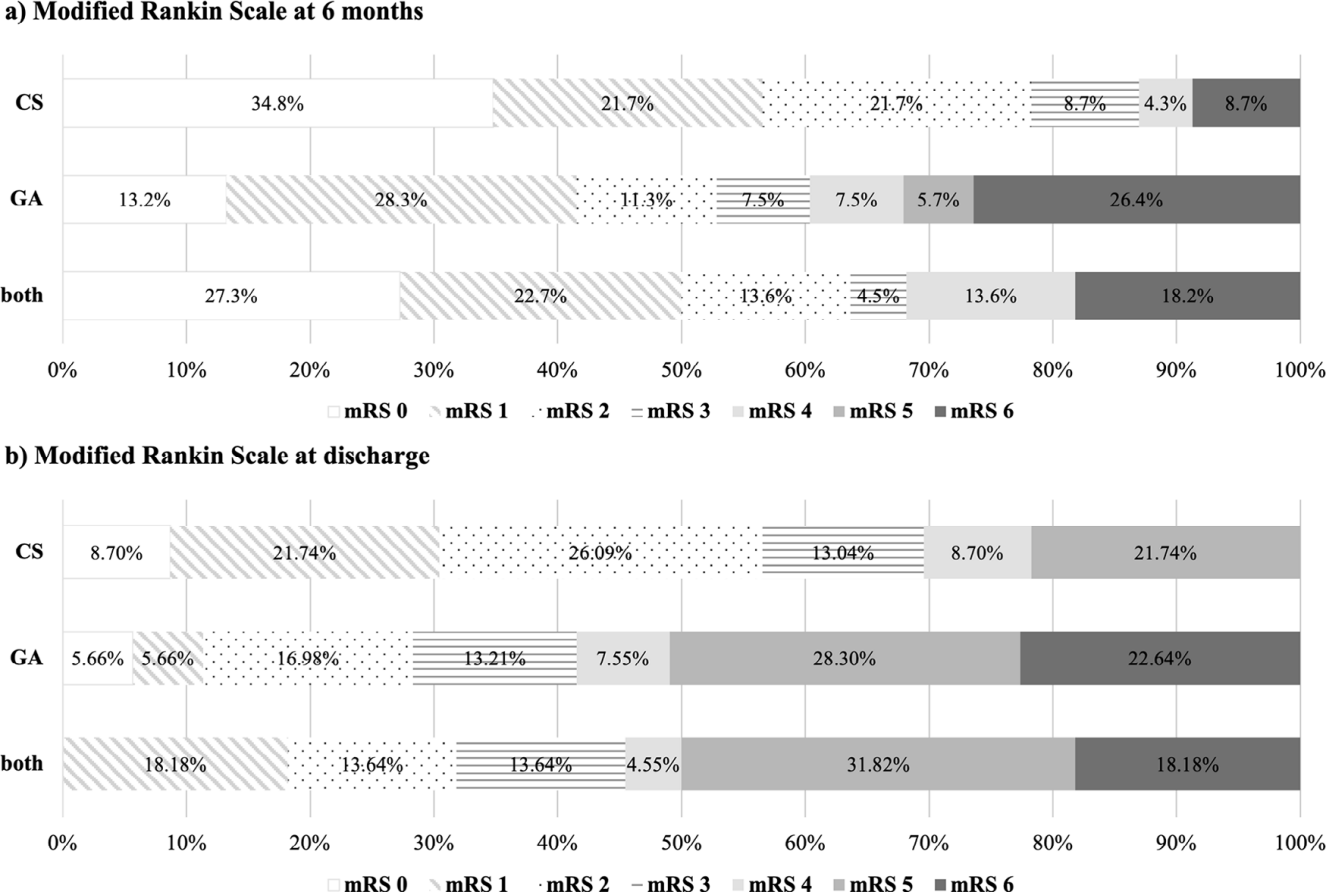
Distribution of modified Rankin Scale (mRS) categories according to anesthesia modality (Conscious Sedation = “CS”, General Anesthesia = “GA” and Conscious Sedation as well as General Anesthesia = “both”). Numbers represent the percentages for each mRS category per group. a) “CS”, “GA” and “both” at six-month follow up. b) “CS”, “GA” and “both” at discharge from hospital.

mRS at six months is displayed in Figure 1a. There was a tendency for a slightly higher percentage of functionally independent patients (mRS 0-2) in the “CS” group (78.3%) at six months. However, this difference did not prove to be statistically significant (p = 0.109). The subgroup analysis comparing single and multiple intra-arterial interventions separately for each anesthesia modality is displayed in Table 2. This analysis also revealed no significant difference in functional outcome at six months between “single CS”, “single GA”, “multiple CS”, “multiple GA” and “both” groups (p = 0.089).

**Table 2.** Subgroup analysis of modified Rankin Scale (mRS) at 6 months and at discharge.

		single CS	multiple CS	single GA	multiple GA	both	p-value
**mRS 6 months**	**mRS 0-2**	11 (68.8%)	7 (100%)	16 (61.5%)	12 (44.4%)	14 (63.6%)	0.089 ^+^
	**mRS 3-6**	5 (31.3%)	0 (0%)	10 (38.5%)	15 (55.6%)	8 (36.4%)	
**mRS discharge**	**mRS 0-2**	9 (56.3%)	4 (57.1%)	9 (34.6%)	6 (22.2%)	7 (31.8%)	0.156 ^+^
	**mRS 3-6**	7 (43.8%)	3 (42.9%)	17 (65.4%)	21 (77.8%)	15 (68.2%)	

Values represent the number of patients with their respective percentages in brackets. mRS: modified Rankin Scale; CS: conscious sedation; GA: general anesthesia.

^+^ Chi-squared test.

### Secondary outcomes

Figure 1b shows mRS at discharge from hospital. This analysis displays no significant difference between “CS”, “GA” and “both” groups (p = 0.056). The subgroup analysis for single and multiple interventions separately also revealed no statistical significance (p = 0.156), as listed in Table 2.

**Figure 2.**
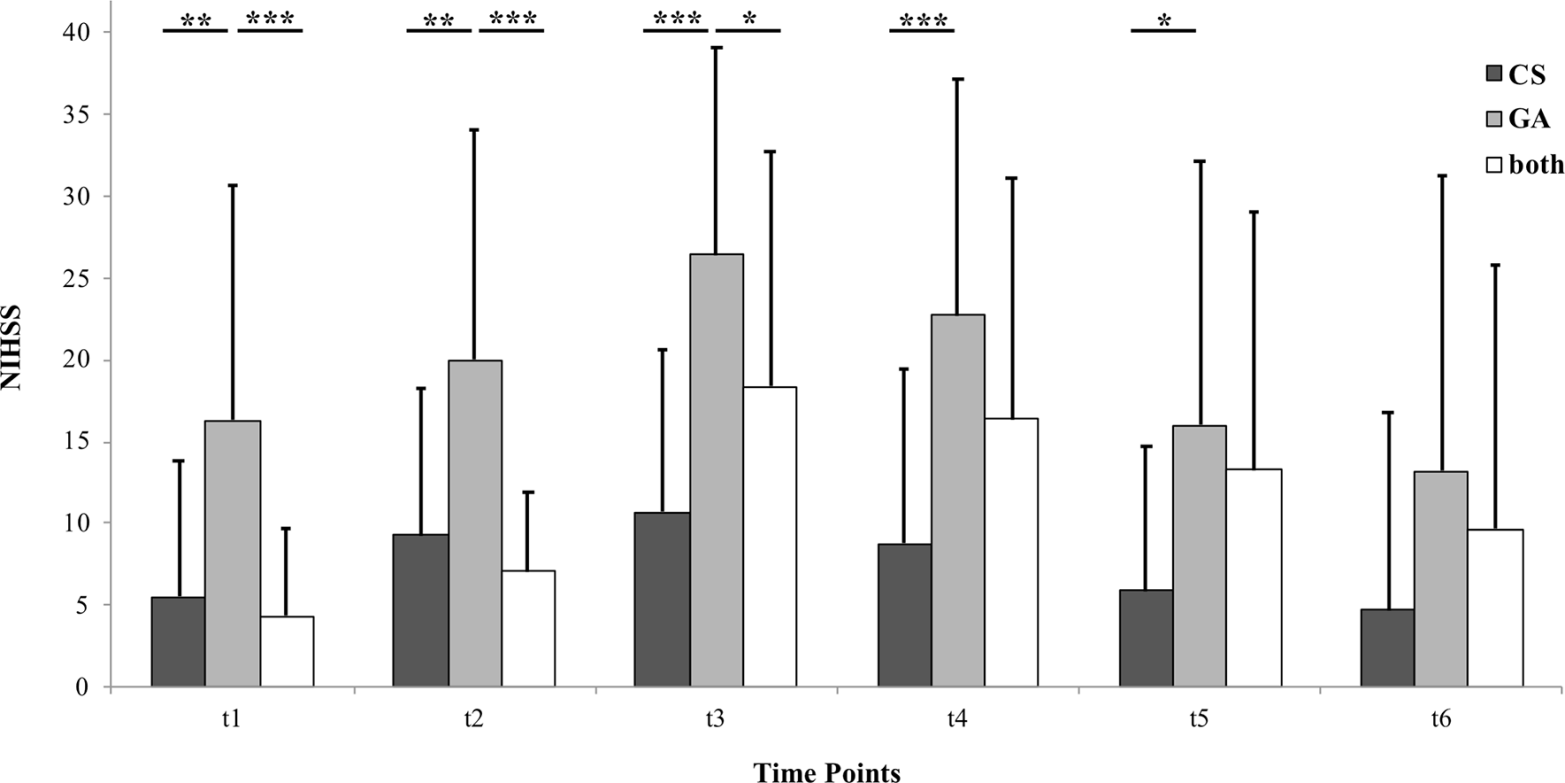
Bar graph depicting National Institutes of Health Stroke Scale (NIHSS) scores of the three anesthesia groups. Patients were treated in conscious sedation (“CS”), general anesthesia (“GA”) or a combination of both (“both”). Each bar represents a different group. Each cluster represents a different time point. Time points are 24 hours before (first) intra-arterial vasodilator intervention (t _1_), immediately before (t _2_), immediately after (t _3_), 24 hours after (last) intervention (t _4_), at discharge from the hospital (t _5_) and at six-month follow up (t _6_). Data are presented as mean + 1 Standard Deviation (SD) as error bars. Significant inter-group differences are highlighted with asterisks. One asterisk represents p < 0.05, two asterisks represent p < 0.01 and three asterisks represent p < 0.001.

The NIHSS time course analysis is presented in Figure 2. ANOVA for repeated measures displayed a significant interaction of “time*treatment” (p = 0.008) and of “time” (p < 0.001). Post-hoc analysis revealed that significant group differences only occur when comparing “GA” to the two other groups. All of these significant differences were between t _1_ and t _5_, meaning between 24 hours before (first) intervention and discharge from the hospital. At the six-month follow up appointment, “CS”, “GA” and “both” did not differ significantly regarding NIHSS.

### Interventional parameters

Interventional parameters are displayed in Table 3. Overall, a total of 237 intra-arterial vasodilator treatments were performed, 65 of which were performed in CS and 172 in GA. Mean duration of intervention was significantly longer if performed in GA (p = 0.002). A total of four interventions had to be excluded from further analysis because of missing data about medication (n = 3) and number of treated arteries (n = 1). Neither choice and dosage of intra-arterial vasodilator, nor number of treated arteries showed significant differences between groups.

**Table 3.** Interventional parameters for intra-arterial vasodilator treatments.

	CS	GA	p-value	Total
**Number of interventions**	65	172		237
**Duration of intervention (min.)**	80 (31)	96 (46)	0.002** ^+^	92 (44)
**Medication (number of interventions)**			0.517 ^x^	
**Nimodipine**	59	147		206
**Papaverine**	3	15		18
**Nimodipine and papaverine**	2	7		9
**Medication dosage (mg)**				
**Nimodipine**	4.8 (1.7)	4.8 (1.7)	0.928 ^#^	4.8 (1.7)
**Papaverine**	200.0 (69)	224.2 (100)	0.696 ^#^	212.1 (94)
**Nimodipine and papaverine**	2.5 (0.07) and 120.0 (0)	3.5 (2) and 123.9 (85)	0.507 ^#^ and 0.953 ^#^	3.0 (1.8) and 121.9 (74)
**Number of treated arteries**	1.8 (0.6)	2 (0.8)	0.057 ^#^	1.9 (0.8)

Where not stated otherwise, values represent the means with the standard deviation in brackets. CS: conscious sedation; GA: general anesthesia.

^+^ Welch’s two sample t-test.

^x^ Chi-squared test.

^#^ Independent samples t-test.

## Discussion

This retrospective study found no significant differences in functionally independent outcomes (mRS 0-2) six months after aSAH in patients who were treated with intra-arterial vasodilators in CS, GA or a combination of both. While NIHSS was significantly higher in patients undergoing endovascular therapy in GA compared to patients of the “CS” or “both” group in the time window 24 hours before intervention up to discharge from the hospital, this difference was no longer found at six months.

To the best of our knowledge, the effect of anesthesia modality (CS versus GA) on functional outcome has not yet been studied for intra-arterial admission of either nimodipine and/or papaverine in patients with refractory CVS after aSAH. Albeit, there have been several research papers published regarding anesthesia in aneurysm treatment. ^21– 23^ Most articles describe both CS and GA to be generally safe for treatment of unruptured aneurysms or aSAH with no clear recommendation for either one.

Additionally, multiple studies have had similar research questions in relation to the choice of anesthesia during endovascular therapy of acute ischemic stroke. Abou-Chebl *et al*. (2010) showed in their analysis of the “North American SOLITAIRE Stent-Retriever Acute Stroke” (NASA) registry, that patients treated in GA experienced poorer neurologic outcome at 90 days and higher mortality rates than patients treated in CS. ^8^ Berkhemer *et al*. (2016) reported similar results in a post-hoc analysis of a prospective trial. ^15^ Correspondingly, a recent analysis of the “Endovascular Therapy Following Imaging Evaluation for Ischemic Stroke 3” (DEFUSE 3) trial by Powers *et al*. (2019) showed higher rates of functional independence (mRS 0-2) and a lower NIHSS score at 24 hours for patients treated in CS. At discharge they did not find a statistically significant difference in NIHSS scores anymore. ^11^

In contrast, most recent randomized controlled trials found a non-inferiority of GA when compared to CS. Hendén *et al*. (2017) reported no difference in outcome at three months or NIHSS after 24 hours in their Anesthesia During Stroke (AnStroke) Trial. ^16^ Schönenberger *et al.* (2016) and Simonsen et al. (2018) report, that GA produced better 3-month outcomes, with the former even finding this result to be significant. ^18, 19^ Finally, a recent meta-analysis determined no significant difference between GA and CS if only randomized controlled trials were considered. ^17^

Our results are in line with these recent randomized controlled trials published for endovascular treatment in acute ischemic stroke. Similar to Schönenberger *et al.* (2016), Hendén *et al*. (2017), Simonsen *et al.* (2018) and Kim *et al*. (2019), we also found no significant difference in functional independency (mRS 0-2) at discharge or six months. ^16– 19^

Similar considerations regarding choice of anesthesia hold true in intra-arterial vasodilator therapy after aSAH and treatment for acute ischemic stroke alike. Disadvantages of GA may be a delay in treatment, hemodynamic changes and complications associated with intubation such as an increased risk for pneumonia. ^8^ Disadvantages of CS may be procedural discomfort for patients, more difficult interventions because of patients’ movements, emergency conversion to GA or increased risk for aspiration. ^8^ Many of the conceived disadvantages of GA have also been analyzed in prospective studies concerning ischemic stroke treatment. For example, Berkhemer *et al.* (2016) as well as Hendén *et al*. (2017) found no treatment delay in the GA group. ^15, 16^ Schönenberger *et al.* (2016) reported no difference in feasibility, safety and intra-interventional complication between the groups. ^18^ They did however discover more postprocedural complications after GA such as delayed extubation and pneumonia. Different authors mention the possibility of blood pressure drops and decreased cerebral blood flow as possible additional complications of GA, which could potentially worsen outcomes because of an increase of the ischemic area. ^15^ Others argue that GA on its own has a neuroprotective effect by lowering the neuronal oxygen need. ^24, 25^ Overall, the similar functional outcome in our study as well as in the above cited studies in ischemic stroke suggest that these factors are of minor relevance.

The major limitations of this study are its retrospective design and the single-center approach. A potential bias could lie in the choice of sedation for treatment. Even before intra-arterial vasodilator treatment, patients who would go on to receive treatments in GA showed significantly higher NIHSS scores when compared to the “CS” and “both” groups. This indicates that patients in clinically and neurologically worse conditions were more likely to be treated in GA. However, this bias was not reflected in our results, as we did not find a significant difference in mRS scores between the anesthesia groups at discharge or at six months and also no significant difference in NIHSS scores at six months. The “GA” group were therefore in an initially worse state but still managed to reach similar outcomes in the long-term clinical course. This suggests to an even greater degree, that GA will not negatively affect long term outcome in these patients.

Furthermore, some subgroups consisted of a small number of patients. The results of this study will have to be replicated by a larger prospective trial in the future.

## Conclusion

Our preliminary results indicate that choice of anesthesia method does not negatively affect six-month outcome in aSAH patients who undergo intra-arterial vasodilator treatment for CVS. Treating physicians should therefore decide between CS and GA individually based on patient characteristics and circumstances.

## Data availability

### Underlying data

Dryad: Functional Outcome after intraarterial vasodilator therapy in CS vs GA, https://doi.org/10.5061/dryad.g4f4qrfq5. ^26^

Data are available under the terms of the Creative Commons Zero “No rights reserved” data waiver (CC0 1.0 Public domain dedication).
