# Supplementary material for: Anesthesia modality does not affect clinical outcomes of intra-arterial vasodilator treatment in patients with symptomatic cerebral vasospasms
Source: F1000Res. 2021 Aug 2;10:417. Originally published 2021 May 25. [Version 2] doi: 10.12688/f1000research.52324.2 (PMC8356260; doi:10.12688/f1000research.52324.2)
Supplement: Peer review history

# Peer review: Reviewer response for version 2

**DOI:** 10.5256/f1000research.59014.r91020

**Published:** 2021-08-09

**Authors:** Giovanna Brandi, Stefan Yu Bögli

**Affiliations:** 1 Institute of Intensive Medicine, Zurich University Hospital, University of Zurich, Zurich, Switzerland

I thank the authors for the changes they made in the revised manuscript and for the answers to our comments.

Is the work clearly and accurately presented and does it cite the current literature?

Yes

If applicable, is the statistical analysis and its interpretation appropriate?

Partly

Are all the source data underlying the results available to ensure full reproducibility?

Yes

Is the study design appropriate and is the work technically sound?

Partly

Are the conclusions drawn adequately supported by the results?

Partly

Are sufficient details of methods and analysis provided to allow replication by others?

Yes

Reviewer Expertise:

Neurocritical care

We confirm that we have read this submission and believe that we have an appropriate level of expertise to confirm that it is of an acceptable scientific standard.

# Peer review: Reviewer response for version 1

**DOI:** 10.5256/f1000research.55588.r88709

**Published:** 2021-07-13

**Authors:** Giovanna Brandi, Stefan Yu Bögli

**Affiliations:** 1 Institute of Intensive Medicine, Zurich University Hospital, University of Zurich, Zurich, Switzerland

This is a retrospective study.

 How were the NIHSS extracted from the patients files? Is NIHSS performed routinely? If the patient was already intubated, how was NIHSS evaluated?

 While aneurysma location as well as Fisher score seem to be similar, WFNS/ H&H seem to differ between CS + GA (e.g. 52% vs 28% WFNS 1). Is there a statistical difference? If so, how do you explain the lack of association thereof with the outcome?

 Patients with high WFNS + Fisher scores commonly remain intubated in case of unsuccessful sedation-reduction trial. How many patients were already under GA before intra-arterial vasodilatation. Is there difference in delay between non-intubated patients to CS or GA intra-arterial vasodilatation?

 When mRS at 6 months is compared between CS and GA only (excluding both) irrespective of if they received a singular or multiple CS/GA statistical significance is reached. How do you explain this finding?

Is the work clearly and accurately presented and does it cite the current literature?

Yes

If applicable, is the statistical analysis and its interpretation appropriate?

Partly

Are all the source data underlying the results available to ensure full reproducibility?

Yes

Is the study design appropriate and is the work technically sound?

Partly

Are the conclusions drawn adequately supported by the results?

Partly

Are sufficient details of methods and analysis provided to allow replication by others?

Yes

Reviewer Expertise:

Neurocritical care

We confirm that we have read this submission and believe that we have an appropriate level of expertise to confirm that it is of an acceptable scientific standard, however we have significant reservations, as outlined above.

# Response

**Published:** 2021-07-24

**Authors:** Corinne Fischer

**Affiliations:** Inselspital, University Hospital Bern, Switzerland

In our institution, NIHSS is routinely assessed in stroke patients at least once a day as well as before and after interventions. In intubated patients, the NIHSS is determined after depth of sedation is decreased as much as clinically possible. The NIHSS assessment is performed according to the recommendation of the National Institute of Neurological Disorders and Stroke ( https://www.ninds.nih.gov/sites/default/files/NIH_Stroke_Scale_Booklet.pdf). Most notably, intubated patients get a default score of 1 for verbally assessed categories such as orientation if they are unable to speak or write. Additionally, aphasia is assessed through writing. Dysarthria is not assessable in the intubated patient.

 The p-values for WFNS, Hunt&Hess, BNI and Fisher scores were added to Table 1. Only the WFNS and Hunt&Hess scores differed significantly between the groups. However, as it has been shown in two earlier publications cited below, WFNS 5 scores at admission are often over-estimated due to accompanying factors e.g. early seizures. In addition, if factors such as intubation, ventilation, sedation, muscle relaxation or insufficient pain stimuli inhibit said motor response, the WFNS grading can be over-estimated while the outcome is not necessarily impacted. Similar effects can alter Hunt&Hess gradings. In contrast the radiologically determined scores (BNI and Fisher) are not influenced by the above factors, which can at least partially explain that these two sores are not significantly different while the clinical ones are. ( Fung *et al.*, 2016; Fung *et al.*, 2015).

 In addition, more severely ill patients corresponding to patients with higher WFNS and Hunt&Hess scores are more probable to undergo intra-arterial vasodilatator therapy in general anesthesia (see also below.)

 Twenty-two patients were already ventilated before the first intra-arterial vasodilator treatment (this corresponds to 37% of all patients who were intubated for the first intra-arterial vasodilator treatment). Unfortunately, due to the retrospective study design, the additional delay of intra-arterial vasodilatation therapy due to intubation was not assessable within the obtained documentation.

 Considering only mRS at 6 months and reducing the analysis to patients who were only treated with one modality (CS or GA), the outcome differed significantly between the two groups (Fisher exact p = 0.044).

 In our opinion this analysis has to be interpreted with caution as the number of patients undergoing intra-arterial vasodilator treatment in CS who showed an unfavorable outcome is very small (n = 5). Furthermore, if the above mentioned patients who remained intubated after the initial bleeding are excluded from this analysis, the result is no longer statistically significant (p = 1.000). This indicates that the difference in outcome of this additional analysis is probably mainly due to the initial disease severity and not due to the anesthesia modality chosen for intra-arterial vasodilator treatment.

# Peer review: Reviewer response for version 1

**DOI:** 10.5256/f1000research.55588.r88125

**Published:** 2021-07-13

**Authors:** Roland Roelz

**Affiliations:** 1 Department of Neurosurgery, Medical Center - University of Freiburg, Faculty of Medicine, University of Freiburg, Freiburg, Germany

This is an interesting preliminary study on the anesthesia modality for endovascular rescue therapies in patients with aneurysmal subarachnoid hemorrhage (aSAH). This single-center retrospective study included 98 aSAH patients (admitted in an 8-year period) selected for endovascular vasospasm therapy. Circa half of these patients were treated in general anesthesia, 25% were treated in conscious sedation and 25% had multiple interventions and both anesthesia modalities were applied. No differences in outcome (6-months mRS) were observed between these groups.

 Carefully interpreted and fully aware of the limitations associated with the study design and data collection, this study may indicate towards safety and non-inferiority of both general anesthesia and conscious sedation for endovascular interventions after aSAH. These findings are in keeping with the pertinent literature on anesthesiologic management of endovascular interventions for stroke.

 I thank the authors for sharing their first experience with conscious sedation as an alternative to general anesthesia for endovascular vasospasm therapy. I encourage investigating the clinical algorithm that triggers the choice for either method. 50% of patients treated in general anesthesia had admission WFNS grades 4 or 5 (i.e. poor grade aSAH) compared to 30% of patients selected for conscious sedation. A fact that may very well explain the presumed tendency towards better outcomes of the latter group. The rate of delayed infarction should have been reported. Given that safety of conscious sedation seems to apply to endovascular vasospasm therapy, future studies are warranted. They should follow a prospective and randomized design. Potential benefits of conscious sedation (i.e. more rapid return to neurological assessability after intervention, less complications of mechanical ventilation etc.) might be of high clinical relevance and could improve outcome of patients who develop clinical vasospasm.

Is the work clearly and accurately presented and does it cite the current literature?

Yes

If applicable, is the statistical analysis and its interpretation appropriate?

Yes

Are all the source data underlying the results available to ensure full reproducibility?

No source data required

Is the study design appropriate and is the work technically sound?

Yes

Are the conclusions drawn adequately supported by the results?

Yes

Are sufficient details of methods and analysis provided to allow replication by others?

Yes

Reviewer Expertise:

Neurosurgery

I confirm that I have read this submission and believe that I have an appropriate level of expertise to confirm that it is of an acceptable scientific standard.

# Response

**Published:** 2021-07-24

**Authors:** Corinne Fischer

**Affiliations:** Inselspital, University Hospital Bern, Switzerland

The following text was added to the methods section of the manuscript: “Choice of anesthesia modality was made by the treating physician on an individual basis. However, according to institutional guidelines, GA was preferred in patients with impaired consciousness (GCS <=8) or insufficient swallowing.”
